# Rbf/E2F1 control growth and endoreplication via steroid-independent Ecdysone Receptor signalling in *Drosophila* prostate-like secondary cells

**DOI:** 10.1371/journal.pgen.1010815

**Published:** 2023-06-26

**Authors:** Aashika Sekar, Aaron Leiblich, S. Mark Wainwright, Cláudia C. Mendes, Dhruv Sarma, Josephine E. E. U. Hellberg, Carina Gandy, Deborah C. I. Goberdhan, Freddie C. Hamdy, Clive Wilson

**Affiliations:** 1 Department of Physiology, Anatomy and Genetics, University of Oxford, Oxford, United Kingdom; 2 Nuffield Department of Surgical Sciences, University of Oxford, Oxford, United Kingdom; Cornell University, UNITED STATES

## Abstract

In prostate cancer, loss of the tumour suppressor gene, *Retinoblastoma* (*Rb*), and consequent activation of transcription factor E2F1 typically occurs at a late-stage of tumour progression. It appears to regulate a switch to an androgen-independent form of cancer, castration-resistant prostate cancer (CRPC), which frequently still requires androgen receptor (AR) signalling. We have previously shown that upon mating, binucleate secondary cells (SCs) of the *Drosophila melanogaster* male accessory gland (AG), which share some similarities with prostate epithelial cells, switch their growth regulation from a steroid-dependent to a steroid-independent form of Ecdysone Receptor (EcR) control. This physiological change induces genome endoreplication and allows SCs to rapidly replenish their secretory compartments, even when ecdysone levels are low because the male has not previously been exposed to females. Here, we test whether the *Drosophila* Rb homologue, Rbf, and E2F1 regulate this switch. Surprisingly, we find that excess Rbf activity reversibly suppresses binucleation in adult SCs. We also demonstrate that Rbf, E2F1 and the cell cycle regulators, Cyclin D (CycD) and Cyclin E (CycE), are key regulators of mating-dependent SC endoreplication, as well as SC growth in both virgin and mated males. Importantly, we show that the CycD/Rbf/E2F1 axis requires the EcR, but not ecdysone, to trigger CycE-dependent endoreplication and endoreplication-associated growth in SCs, mirroring changes seen in CRPC. Furthermore, Bone Morphogenetic Protein (BMP) signalling, mediated by the BMP ligand Decapentaplegic (Dpp), intersects with CycD/Rbf/E2F1 signalling to drive endoreplication in these fly cells. Overall, our work reveals a signalling switch, which permits rapid growth of SCs and increased secretion after mating, independently of previous exposure to females. The changes observed share mechanistic parallels with the pathological switch to hormone-independent AR signalling seen in CRPC, suggesting that the latter may reflect the dysregulation of a currently unidentified physiological process.

## Author summary

Sex hormones, like testosterone, are steroids required for development and maintenance of reproductive organs, like the prostate, and for growth of prostate cancer in its early stages. However, when cancer patients are given treatments that block production of these steroids, some resistant cells survive and grow, inevitably producing an untreatable and lethal form of the disease. Frequently, these resistant cells still require the steroid receptor, but unlike in normal cells, this receptor works in the absence of hormone. Loss of the human tumour suppressor gene, *Retinoblastoma*, is often critical in driving this process. Here, we study a prostate-like cell in the fruit fly, in which we have discovered that a steroid receptor can normally drive growth in the absence of hormone after flies have mated. We find that this switch to steroid-independence is controlled by the fly equivalent of *Retinoblastoma*. We also identify ways to suppress this switch by targeting other genes involved in prostate cancer. Our work therefore suggests the switch to hormone-independent prostate cancer growth in cancer patients may reflect the mis-regulation of a normal physiological process found in flies and humans, and provides clues for the development of treatments that might prevent emergence of human therapy-resistant tumours.

## Introduction

The secretory, paired male accessory gland (AG) is the major seminal fluid-producing organ in the fruit fly *Drosophila melanogaster*. It shares several functional similarities with the prostate and the seminal vesicles, which constitute the main mammalian accessory glands [1–5]. The AG is formed from a monolayer epithelium consisting of two distinct octoploid and binucleate cell types: main cells (MCs) (~1000 cells/AG lobe) and secondary cells (SCs) (~40 cells/AG lobe) [6]. The prostate gland and the SCs, unlike the MCs, increase in size as adults age [7]. Upon repeated mating, all SCs grow more rapidly, although about 25% of SCs grow more than the other cells, because they undergo genome endoreplication. This process, which does not lead to cell division, increases SC secretory activity and therefore enhances replenishment of the AG luminal contents [2].

**Fig 1:**
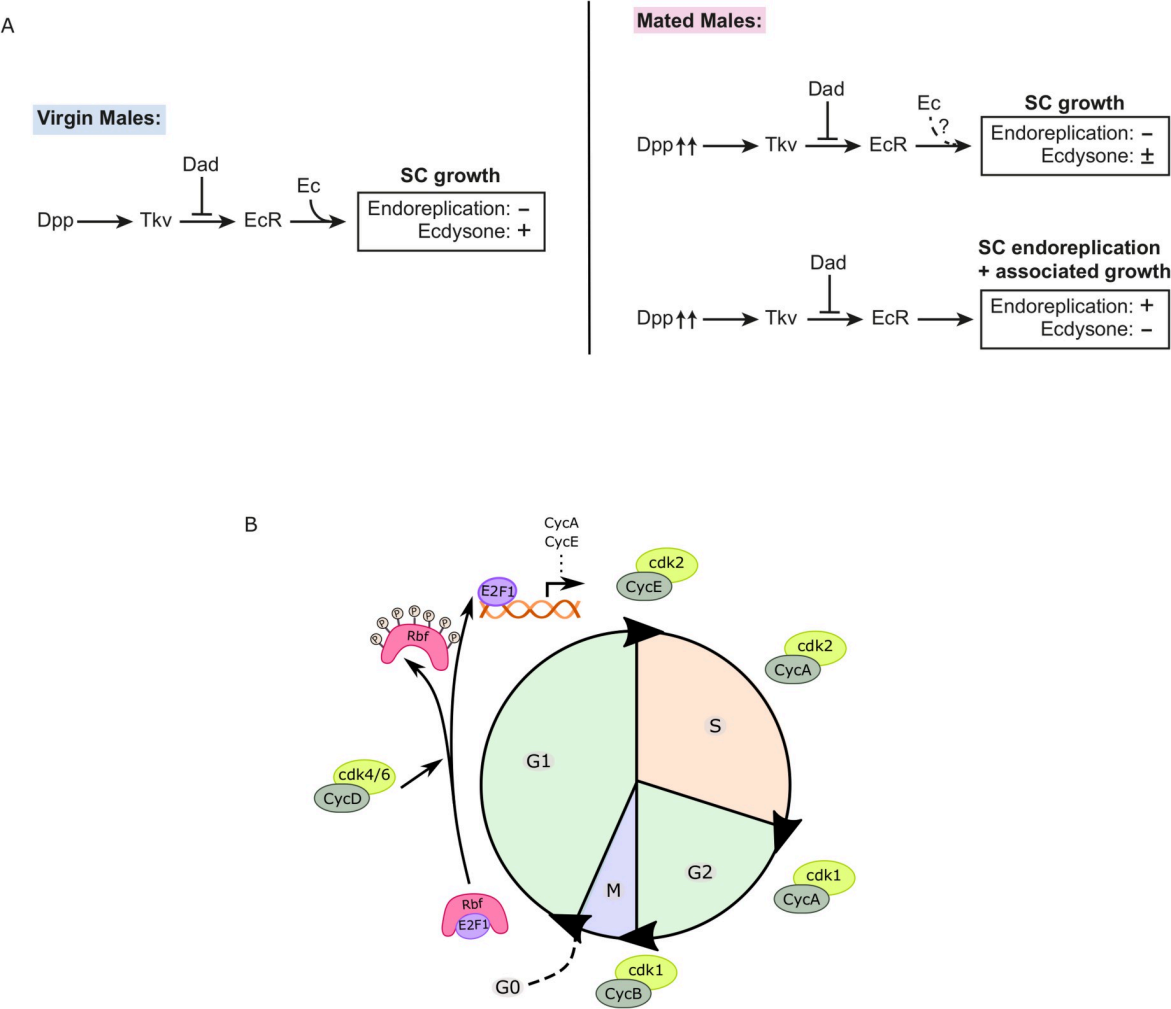
Endoreplication-associated SC growth in mated male *Drosophila* is regulated by hormone-independent EcR signalling. (A) Schematic showing model of growth regulation in *Drosophila* SCs, as measured by nuclear growth^2^. Growth in virgin males is controlled by hormone-dependent EcR signalling, which itself is stimulated by BMP signalling involving the ligand Decapentaplegic (Dpp) and the BMP type I receptor Thick Veins (Tkv) and inhibited by the transcriptional repressor Dad. After mating, 25% of cells endoreplicate and grow via an EcR-mediated mechanism that does not require hormone. The remaining 75% of cells also increase their growth, and most of this growth also appears to involve hormone-independent EcR signalling under the control of autocrine BMP signalling stimulated by increased secretion during mating. (B) Schematic describing key features of the cell cycle transitions and the roles of CycD, Rb, E2F1 and CycE in driving cells into S phase. Rb is a negative regulator of the cell cycle. In its active form, Rb binds to the E2F1 transcription factor and inhibits its activity. When Rb is inactivated by hyperphosphorylation, E2F1 transcribes genes, including CycE. CycD/cdk4/6 mediates events that occur in early G1-phase (including Rb/E2F1 regulation) and CycE/cdk2 is required for initiating the G1/S transition via a negative feedback loop that inhibits Rb activity, while Cyclin A (CycA)/cdk2, CycA/cdk1 and Cyclin B (CycB/cdk1) are involved in driving cells through the rest of the cell cycle.

The Decapentaplegic (Dpp) signalling pathway, orthologous to the mammalian bone morphogenetic protein BMP2/BMP4 pathway, and the fly steroid receptor, the Ecdysone Receptor (EcR), play essential roles in regulating SC nuclear growth, a proxy for cell growth (and hereafter referred to as ‘growth’), and endoreplication, specifically in adult males [2,7]. In virgin males, the steroid hormone, 20-hydroxyecdysone (20-HE or ‘ecdysone’), is required for growth. In mated males, however, SC endoreplication and associated growth is ecdysone-independent, while there is also enhanced endoreplication-independent growth, much, if not all, of which also appears to be independent of hormone [2] (Fig 1A). In the absence of 20-HE, when males are multiply mated, SC growth is unaffected, even though in virgins, it is strongly inhibited, demonstrating that these different forms of growth control can be uncoupled by analysing multiply mated versus virgin males.

One potential explanation for the switch to hormone-independence is that because the levels of ecdysone increase in males exposed to previously mated females [8], the change allows rapid EcR-dependent growth to take place irrespective of female exposure. Autocrine BMP signalling, stimulated by increased secretion from SCs during mating [4], elevates EcR protein levels and hormone-independent activation, promoting endoreplication in SCs [2]. It remains unclear whether other signalling pathways play important roles in the switch to ecdysone-independent EcR activity.

During the early stages of prostate cancer, tumour growth requires the androgenic steroids and the androgen receptor (AR). Although androgen-deprivation therapy (ADT), a mainstay treatment for advanced prostate cancer, has an initial response rate of 90%, within two years, most cases have inevitably progressed to an aggressive and incurable form of prostate cancer, castration-resistant prostate cancer (CRPC) [9–11]. Several processes play a role in the development of CRPC. In most cases, AR signalling remains essential for the maintenance and progression of the tumour, although, as is also found in SCs after mating, its activation no longer requires androgens [9]. The molecular basis of this AR-controlled growth can involve *AR* gene amplification or increased expression, AR mutations, AR signalling pathway changes, or AR cofactors leading to androgen hypersensitivity or constitutive AR activity [9,12]. Changes in upstream BMP-6 signalling have also been reported to promote hormone-independent AR activity [11].

Loss of the tumour suppressor gene *Retinoblastoma* (*Rb*) is commonly observed in several cancer types and is often considered essential for the early development of cancer [10,13]. However, in prostate cancer, *Rb* loss is associated with late-stage prostate cancer progression [10]. Rb negatively regulates proteins in the E2F transcription factor family, which are involved in activating genes that are essential for the progression of the Synthesis (S)-phase of the cell cycle, including Cyclin E (CycE) [14,15]. During Gap1 (G1)-phase, Rb is gradually phosphorylated, first by the growth factor-stimulated Cyclin D (CycD)/cyclin dependent kinase (cdk) 4/6 complex, then by CycE/cdk2 in late G1 (Fig 1B). It remains hyperphosphorylated until the Mitotic (M)-phase. Hyperphosphorylation of Rb changes its conformation and releases E2F factors, thereby enabling E2F-dependent transcriptional activity [14].

In early prostate cancer, Rb tightly regulates E2F1, which in addition to acting on *CycE*, also controls the expression levels of *AR*, therefore linking Rb/E2F and AR signalling. Loss of *Rb* during cancer progression leads to an unsupervised activation of E2F, thereby increasing the protein levels of AR [10]. This promotes prostate cancer cellular growth and proliferation, even after ADT, at least partly explaining the link between loss of *Rb* and CRPC [10,16].

Since endoreplicating cells utilise a variant of the cell cycle machinery to drive DNA replication [17,18], we investigated the molecular mechanisms controlling ecdysone-independent, EcR-mediated endoreplication in SCs, focusing particularly on whether Rbf, the *Drosophila* Rb homologue, might be involved. Here we demonstrate that CycD, Rbf and E2F1 are essential for SC endoreplication and growth after mating, with Rbf, and in some cases E2F1, playing an additional role in controlling binucleation of SCs. CycD/Rbf/E2F1 signalling regulates EcR levels and is strongly dependent on the EcR, but not ecdysone, to control endoreplication, while EcR requires CycE to fully promote endoreplication. Our data therefore reveal a physiological switch that is triggered by mating and involves Rbf/E2F1-activated control of hormone-independent EcR-mediated endoreplication and growth, thus mirroring pathological growth-promoting changes in AR signalling associated with Rb loss in CRPC.

## Results

### Activated Rbf suppresses binucleation in SCs

To test the effect of the cell cycle regulators, CycD, Rbf, E2F1 and CycE, on SC growth and endoreplication, we initially either overexpressed these molecules or RNAis targeted against them in adult SCs. To do this, we used the esg^ts^F/O driver system, which induces overexpression of transgenes specifically in adult SCs of the AG under the control of the yeast GAL4 transcription factor [2,7]. This line ubiquitously expresses a temperature-sensitive form of the GAL4 inhibitor GAL80, which blocks *esg*-GAL4-dependent transgene expression until the temperature is shifted to 28.5°C, which inhibits GAL80 function. Newly eclosed males were switched from 25°C to 28.5°C for 6 days to express the transgene in adult SCs, but not during their development.

**Fig 2:**
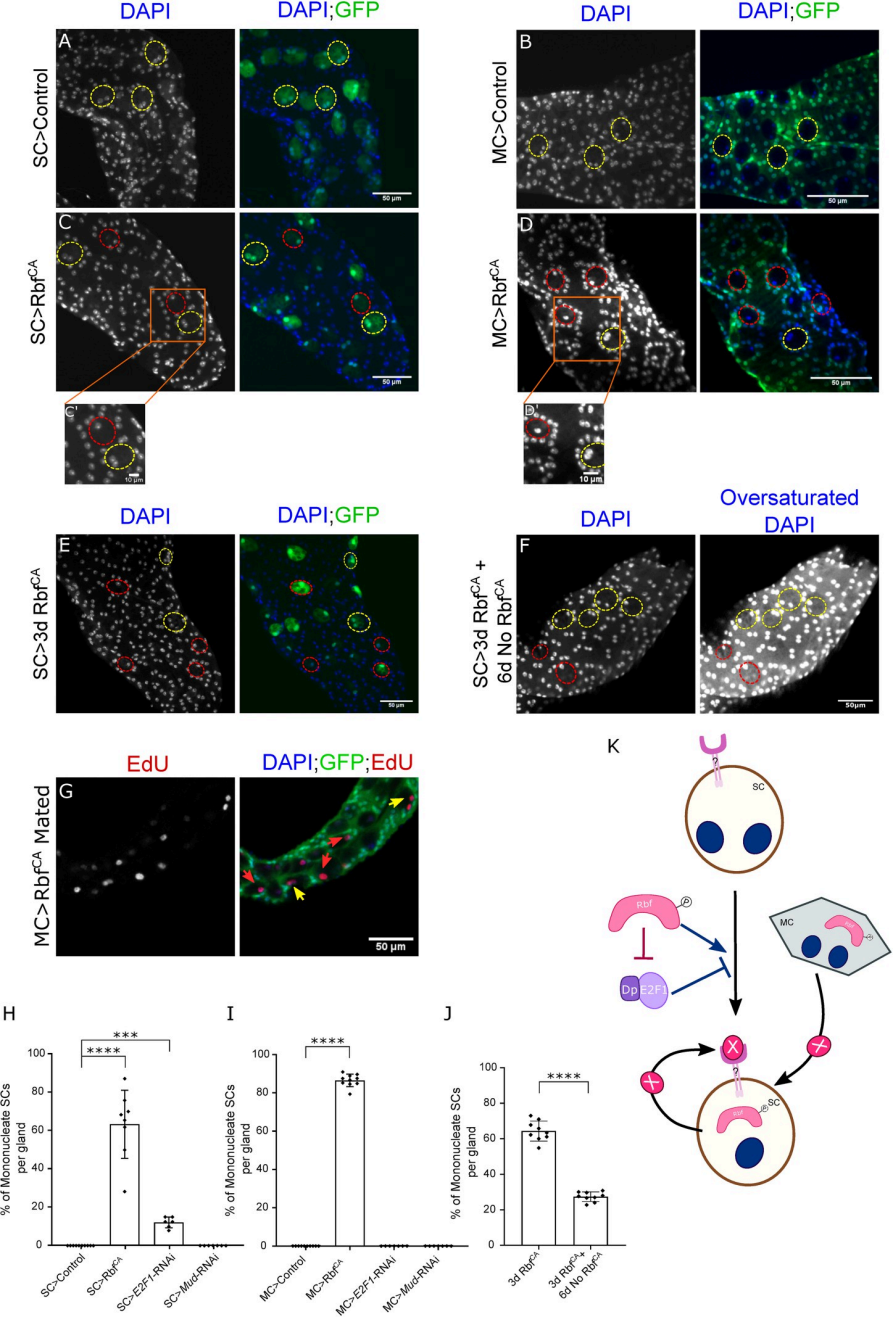
The Rbf/E2F1 axis maintains adult SC binucleation state. (A-G) Nuclei are stained with DAPI (blue). Dashed red ellipses mark the outline of mononucleate SCs; dashed yellow ellipses mark the outline of binucleate SCs; red arrows mark the nuclei of mononucleate SCs; yellow arrows mark the nuclei of binucleate SCs. (A-D) Images show distal tip of AGs from 6-day-old adult virgin males of controls or of flies expressing Rbf^CA^ either under the control of the esg^ts^F/O driver (A, C; SC driver) or the *Acp26Aa*-GAL4 driver (B, D; MC driver), which activates nuclear GFP production (also stains cytoplasm in A, C). Orange boxes outline the area zoomed in the insets C’, D’. (E) Image shows distal tip of an AG from a 3-day-old adult virgin male expressing Rbf^CA^ in SCs under esg^ts^F/O control. (F) Image shows distal tip of an AG from a 9-day-old virgin male, which expressed Rbf^CA^ in SCs for 3 days following eclosion, and was then incubated for 6 days at 25°C to block further Rbf^CA^ expression. No GFP was expressed during the 6-day follow-up period, so SCs were identified using their well-characterised vacuolar morphology ^2^. (G) Image shows the distal tip of an AG of a 6-day-old multiply-mated male expressing Rbf^CA^ under the control of the *Acp26Aa*-GAL4 driver. The AG was stained for EdU presence, revealing that mononucleate SCs are still able to endoreplicate. (H-J) Bar charts depicting the mean % of mononucleate SCs per gland of virgin males in controls or males expressing transgenes either in SCs (H, J) or MCs (I). Expression of Rbf^CA^ either in SCs or MCs induces SC mononucleation in a reversible manner, but this transgene has no effect in MCs. Knocking down *E2F1* in SCs promotes SC mononucleation but no effect is observed when knocked down in MCs. Kruskal Wallis test; Dunn’s post hoc test; n≥6 glands (H-J). (K) Schematic detailing nucleation state regulation in adult SCs. Expression of a constitutively active form of Rbf, Rbf^CA^, which has mutations in three of its four CDK phosphorylation sites, in SCs is able to induce mononucleation. This phenotype is negatively regulated by E2F1. Additionally, expression of Rbf^CA^ in MCs is able to activate mononucleation in SCs. In one model, it might induce secretion of ‘X’, which could activate an unknown receptor (?) that drives SC mononucleation; alternatively, Rbf^CA^ may suppress secretion of a molecule, which promotes binucleation. It is possible that Rbf^CA^ when expressed in SCs also at least partly promotes mononucleation via the same mechanism. Scale bars correspond to 50 μm (A-G) and 10 μm (C’, D’). 0.0001≤***p≤0.001; ****p≤0.0001.

A surprising effect on SCs was observed when we expressed a constitutively activated form of Rbf, Rbf^CA^, which has three of its four phosphorylation sites substituted, thereby making it refractory to regulation by CycE/Cdk2 and CycD/Cdk4 [19]. This resulted in 63 ± 12% of SCs becoming mononucleated after 6 days of adult expression (Fig 2C and 2C’); control glands never contain mononucleate SCs (Fig 2A, and bar chart in Fig 2H). This was not simply the result of binucleate cells dividing to form two mononucleate cells, because the number of SCs remained unchanged (40.0 ± 5.1 in controls and 40.8 ± 2.2 in Rbf^CA^-expressing cells). Remarkably, we observed that the mononucleate phenotype could be partially reversed to a binucleate state, when the expression of Rbf^CA^ was blocked again by reducing the temperature to 25°C to reactivate GAL80 (compare Fig 2F with Fig 2E, and see bar chart in Fig 2J). To test whether inhibition of E2F1 is involved in this phenomenon, the *E2F1* gene was knocked down in SCs, resulting in 12 ± 3% of SCs becoming mononucleated (Fig 2H; data in S1A Fig). Since mononucleation is never observed in controls, this suggests that E2F1 inhibition plays a role in Rbf-induced mononucleation, and E2F1 is involved in normal maintenance of binucleation in SCs.

In order to determine whether the nucleation state of binucleate MCs is also affected by Rbf, Rbf^CA^ was overexpressed in these cells, using a GAL80^ts^-regulated *Acp26Aa*-GAL4 MC driver (compare Fig 2B with Fig 2D and 2D’, bar chart in Fig 2I) [20,21]. Surprisingly, there was no effect on MC binucleation, but 86 ± 3% SCs became mononucleated. These SCs could still endoreplicate upon mating (Fig 2G), as assayed by nuclear incorporation of 5-ethynyl-2′-deoxyuridine (EdU), a synthetic analogue of thymidine delivered in the food. This demonstrates that adult endoreplication is not affected by the nucleation state of SCs and that the mononucleation phenotype is unlikely to be mediated by transfer of endoreplication-inhibiting Rbf^CA^ to SCs. This effect of Rbf^CA^ could not be explained by leaky expression of the *Acp26Aa*-GAL4 driver in SCs, because no expression of a UAS-controlled nuclear GFP could be detected in SCs with this driver (Fig 2D), yet the proportion of mononucleate SCs induced was higher than when Rbf^CA^ was highly expressed in SCs (Fig 2H and 2I). Expression of *E2F1*-RNAi in MCs did not induce mononucleation either in SCs or MCs (Fig 2I and data in S1B Fig).

In summary, these results suggest that Rbf may control SC nucleation state in two different ways (Fig 2K); first, through paracrine regulation, which may not require E2F1 inhibition, and where an unknown downstream signal (eg. X in Fig 2K) is involved; second, via cell-autonomous regulation, where E2F1 appears to be partly involved, and which could involve autocrine X and/or another intracellular mechanism.

### EcR-induced SC endoreplication is at least partly dependent on CycE

Our previous work [2] demonstrated that SC growth is regulated via three different mechanisms (Fig 1A): (i) SCs grow during ageing of adult virgin males (mating-independent growth), (ii) after multiple matings, all SCs increase their growth, even in the absence of endoreplication (mating-dependent, endoreplication-independent growth), and (iii) approximately 25% of SCs undergo endoreplication after multiple matings, which promotes additional endoreplication-associated growth (mating-dependent, endoreplication-associated growth). The latter two mechanisms appear to be primarily EcR-dependent, but hormone- (ecdysone-) independent. By measuring SC growth and endoreplication in virgin and multiply mated males in our current study, we assessed how different genetic manipulations modulated mating-dependent versus -independent growth. However, it was typically not possible to clearly distinguish effects on the two mating-dependent forms of growth. This is because in our experiments, some SCs are likely to endoreplicate at least in part because they are expressing higher levels or different ratios of transgenes than non-endoreplicating cells, and this could presumably influence endoreplication-independent growth as well as endoreplication-associated growth.

CycE has been implicated in the regulation of endoreplication in several *Drosophila* cell types, including SCs [2,22]. Using the esg^ts^F/O driver system, we confirmed that *CycE* knockdown resulted in a decrease in SC growth in mated males, but also in virgin males, where essentially no endoreplication takes place (compare S2A and S2B Fig with S2C and S2D Fig and bar chart in S2F Fig). Furthermore, consistent with previous observations [2], *CycE*-RNAi expression completely inhibited mating-dependent endoreplication normally seen in 25% of SCs (S2A–S2D Fig, bar chart in S2E Fig). Contrary to our previous findings [2], SC growth in *CycE*-knockdown cells was significantly increased after mating (S2F Fig), although this change was only detectable because of the reduced number of genotypes being compared in our current statistical analysis. CycE is therefore required for SC growth in virgin and mated males, and for mating-dependent endoreplication, but some endoreplication-independent growth in mated males does not appear to be mediated by this molecule.

**Fig 3:**
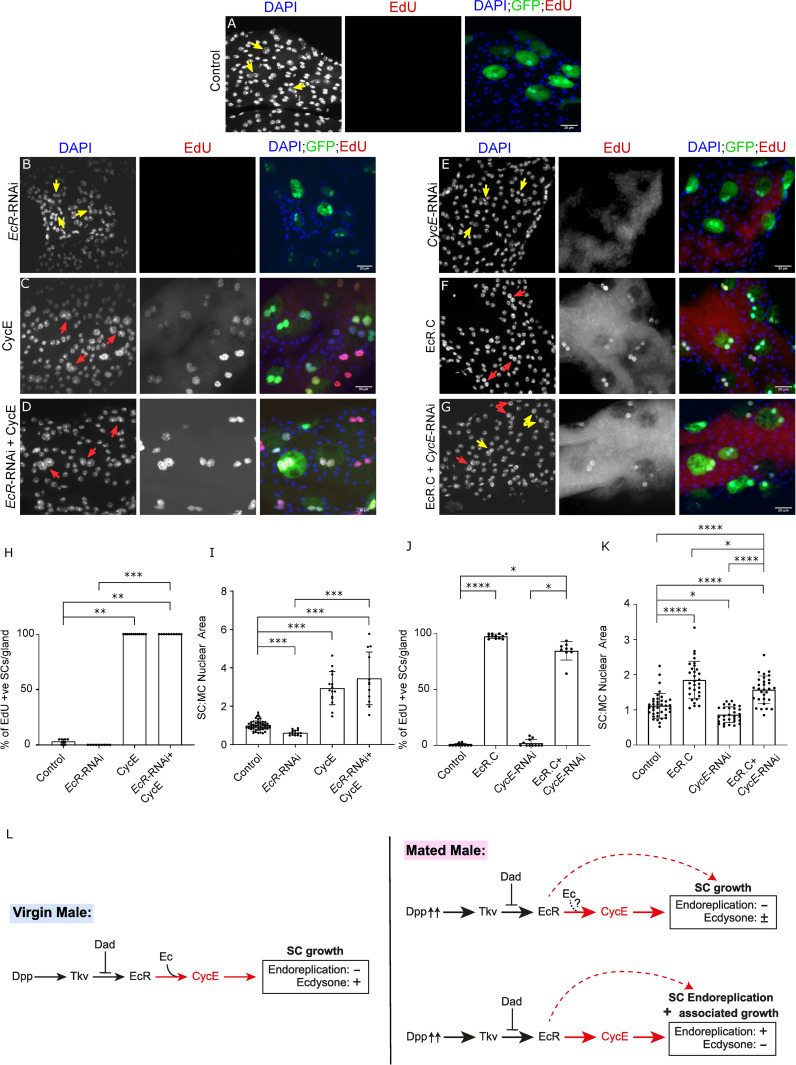
EcR-induced SC endoreplication is at least partly dependent on CycE. (A-G) Images show distal tip of AGs from 6-day old adult virgin males expressing nuclear GFP alone (control; this also stains the cytosol) or in combination with additional transgenes in SCs, listed in individual images, under the control of the esg^ts^F/O driver and stained for EdU incorporation in SCs. Nuclei are stained with DAPI (blue). Red arrows point to pairs of EdU^+^ SC nuclei and yellow arrows point to pairs of EdU^-^ SC nuclei for each transgene. (H-K) Bar charts depicting the mean % of EdU^+^ SCs per gland (H, I) and the mean ratio of the size of SC nuclei relative to neighbouring MC nuclei (J, K) for virgin males expressing the different combinations of transgenes in SCs. Knocking down *EcR* does not affect CycE-mediated SC growth and endoreplication (D, H, I). *CycE* is at least partly required for EcR-C-induced SC growth and endoreplication (G, J, K). (L) Schematic showing that in SCs, *EcR*’s effects on endoreplication and growth are at least partly regulated by *CycE* (new finding highlighted in red); previous experiments^2^ have shown that CycE is also involved in hormone-dependent SC growth in virgin males and endoreplication-independent growth in mated males, much of which is hormone-independent. One-way ANOVA; Sidak’s multiple comparisons test; n≥10 glands (H). Kruskal Wallis test; Dunn’s post hoc test; n≥9 glands (I). Welch ANOVA; Games-Howell post hoc test; n≥15 cells (J). Kruskal Wallis test; Dunn’s post hoc test; n≥8 glands (K). Scale bars correspond to 20 μm. The error bars show the standard deviation within the sample. 0.01<*p<0.05; 0.001<**p≤0.01; 0.0001<***p≤0.001; ****p≤0.0001.

Since we had previously shown that the EcR is necessary and sufficient to induce endoreplication in SCs, we investigated whether CycE and EcR genetically interact with each other to regulate this process. When *CycE* was co-overexpressed with *EcR*-RNAi, SCs behaved very similarly to cells expressing *CycE* alone (compare Fig 3D with Fig 3C). 100% of SCs endoreplicated (compare Fig 3D to Fig 3A–3C, bar chart in Fig 3H), and CycE-induced overgrowth was not inhibited (Fig 3A–3D and 3I). Since *EcR*-RNAi expression suppresses endoreplication in mated males and inhibits growth of SCs in virgin and mated males, these observations suggest that CycE-dependent effects are not mediated by the EcR.

When *EcR-C*, a synthetic EcR isoform with a deleted N-terminal domain that induces high levels of endoreplication when expressed in SCs, was co-expressed with *CycE*-RNAi, 85 ± 8% of SCs endoreplicated as compared to 98 ± 2% of SCs when only EcR-C was expressed (compare Fig 3G to Fig 3E and 3F, bar chart in Fig 3J). There was no overlap in these data (Fig 3J), suggesting that CycE is at least partly required for EcR-C-stimulated endoreplication. However, the decrease in percentage of endoreplicating SCs was not significant, which may be attributed to the lower statistical power of the Dunn’s post-hoc test used to analyse these data. EcR-C-induced SC growth was partially, but significantly, inhibited by knockdown of *CycE*, although this growth was still significantly higher than in control SCs (Fig 3A, 3E–3G and bar chart in Fig 3K). These results suggest that at least some of the high-level SC endoreplication and growth induced by EcR-C requires CycE. However, the remainder may not require CycE or may still occur in the presence of low levels of CycE (Fig 3L). This is in contrast to normal, mating-induced EcR-dependent endoreplication, for which CycE is essential.

### CycD, Rbf and E2F1 are essential for the regulation of SC endoreplication and growth

**Fig 4:**
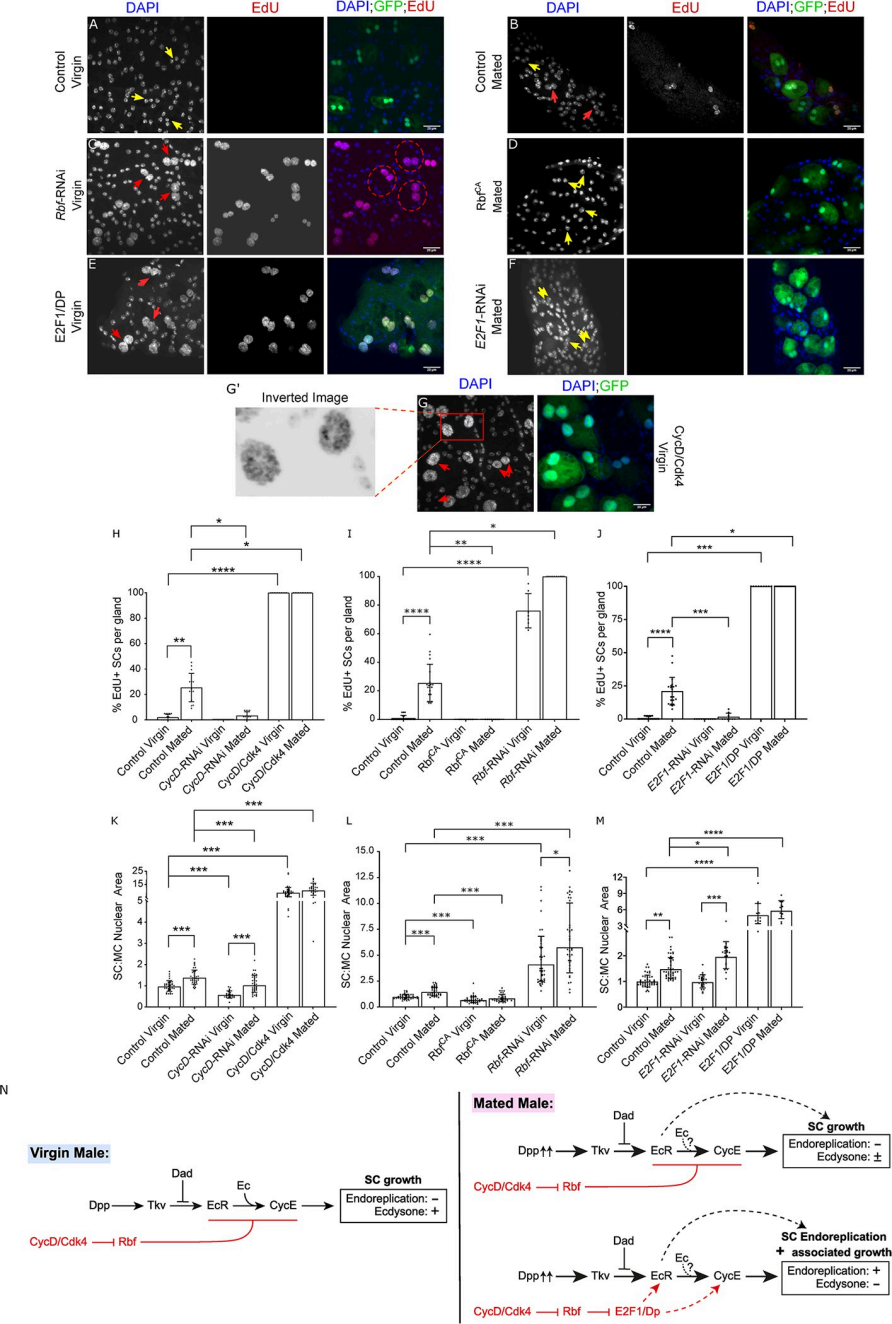
Cell cycle components regulate SC endoreplication and growth. (A-G) Images show distal tip of AGs from 6-day-old adult virgin (A, C, E, G) or multiply mated (B, D, F) males expressing nuclear GFP alone (control; this also stains the cytosol; A, B) or in combination with additional transgenes in SCs under the control of the esg^ts^F/O driver and stained for EdU incorporation. Nuclei are stained with DAPI (blue). Red arrows point to EdU^+^ pairs of SC nuclei and yellow arrows point to EdU^-^ pairs of SC nuclei for each transgene (or in some cases, single nuclei when Rbf^CA^ is expressed). (G’) Magnified negative image of orange square in image G to highlight the polytene-like nuclei in SCs overexpressing CycD/Cdk4. (H-M) Bar charts depicting mean % of EdU+ SCs per gland (H-J) and the geometric mean ratio of the size of SC nuclei relative to neighbouring MC nuclei (K-M) of virgin and mated control males and males expressing different transgenes in SCs. CycD is necessary and sufficient to promote normal SC growth and endoreplication (H, K, and S2I and S2J Fig). Rbf negatively regulates SC growth and endoreplication in virgin and mated males (C, D, I, L). E2F1 is not necessary for SC growth in virgins, but in mated males, it appears that overall it is required for endoreplication, but negatively regulates endoreplication-independent growth (E, F, J, M). (N) Schematic showing that *CycD* and *Rbf* control SC endoreplication and associated growth via *E2F1* in mated males, but for endoreplication-independent growth in mated males and for growth in virgin males, E2F1 does not appear to be involved (new findings shown in red). Kruskal Wallis test; Dunn’s post hoc test (H-J). Welch ANOVA on log-transformed data; Games-Howell post hoc test (K-M). n≥9 glands (H); n≥7 glands (I); n≥9 glands (J); n≥24 glands (K); n≥33 cells (L); n≥12 cells (M). Scale bars correspond to 20 μm. The error bars show the standard deviation (H-J) and geometric standard deviation (K-M) within the sample. 0.01<*p<0.05; 0.001<**p≤0.01; 0.0001<***p≤0.001; ****p≤0.0001.

As well as observing a function for *Rbf* and *E2F1* in SC binucleation, we also found a role for these genes and their upstream cell cycle regulator CycD in SC growth and endoreplication. Knocking-down *CycD* in SCs abolished mating-dependent endoreplication and inhibited SC growth in virgin and mated males (see bar charts in Fig 4H and 4K; data in S2J and S2J’ Fig versus S2H and S2H’ Fig). However, similar to when *CycE* was knocked down, we observed that expression of *CycD*-RNAi did not fully suppress all endoreplication-independent SC growth after mating, because *CycD*-knockdown SCs from mated males were larger than those from virgin males (Fig 4K). To test the effect of increased CycD activity, CycD was overexpressed with its kinase partner, Cdk4, in SCs (Fig 4G). All SCs endoreplicated extensively, resulting in polytene-like chromosomes in both virgin and mated males, and significantly increased growth (Figs 4G’ and S2I versus S2G Fig, bar charts in Fig 4H and 4K and EdU incorporation in S2I’ Fig). Taking these results together, we conclude that CycD is required to induce ecdysone-independent SC endoreplication in mated males. Furthermore, CycD regulates ecdysone-dependent SC growth in virgin males and some of the ecdysone-independent growth observed after mating. CycD overexpression is sufficient to drive endoreplication and growth in unmated males.

Overexpression of Rbf^CA^ suppressed mating-dependent endoreplication and all SC growth in virgin and mated males (compare Fig 4D and 4B, bar charts in Fig 4I and 4L). In these experiments, the SC nuclear growth for mononucleate SCs was calculated as the ratio of the area of the single nucleus to the total nuclear area of both nuclei in the adjacent MC area. Consistent with the reduction in nuclear growth, we also observed a reduction in the total SC area of Rbf^CA^-expressing SCs (S3 Fig), supporting our conclusion that reduced overall nuclear area was not just the consequence of fusing two nuclei into a single nucleus. Expressing *Rbf*-RNAi resulted in an increase in endoreplication and growth in all SCs in both virgin and mated males (compare Fig 4C and 4A, bar charts in Fig 4I and 4L). Hence, Rbf normally limits SC endoreplication and growth, and its hyperactivation suppresses all forms of SC growth in both virgins and mated males.

To increase E2F1 activity in SCs, it was co-expressed with its dimerization partner, Dumpy (DP). Increased SC growth was observed and all SCs underwent endoreplication in both virgin and mated males (Fig 4E, bar charts in Fig 4J and 4M). *E2F1* knockdown suppressed all endoreplication after mating (Fig 4F, bar charts in Fig 4J). Surprisingly, however, knockdown had no effect on SC growth in virgin males and growth was increased in mated males versus mated controls (Fig 4M). Therefore, E2F1 is not necessary for endoreplication-independent SC growth in virgin and mated males. Indeed, since knockdown cells in mated males have larger nuclei than controls after mating, E2F1 appears to partially suppress endoreplication-independent growth that occurs after mating, as well as driving endoreplication and presumably endoreplication-associated growth. Knockdown of the other Rbf-regulated *E2F* gene in *Drosophila*, *E2F2*, had no effect on SC growth and endoreplication (S4 Fig), suggesting that this gene is not involved in these processes.

We conclude that the cell cycle components CycD, Rbf and E2F1 all play important roles in the regulation of adult SC endoreplication and associated growth, although CycD/Rbf do not seem to act via E2F1 to control endoreplication-independent SC growth either in virgin males, which is regulated by ecdysone, or in mated males, which is ecdysone-independent (Fig 4N).

### BMP signalling is downstream of CycD, but upstream of E2F1 in the regulation of EcR expression, endoreplication and growth in SCs

**Fig 5:**
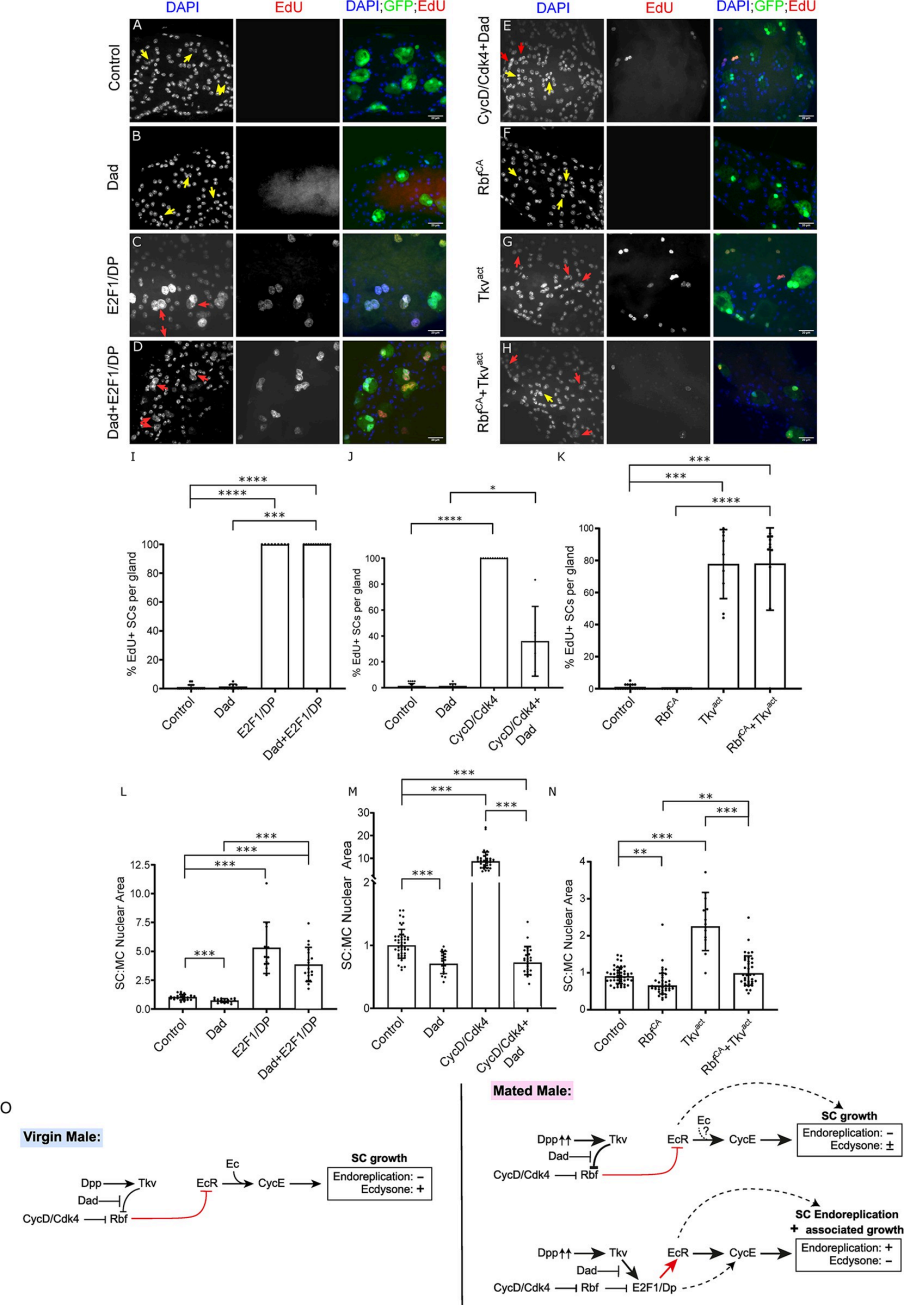
Interaction between CycD/Rbf/E2F1 and BMP signalling in the regulation of SC endoreplication and growth. (A-H) Images show distal tip of AGs from 6-day-old adult virgin males expressing nuclear GFP alone (control; this also stains the cytosol; A) or in combination with additional transgenes in SCs under the control of the esg^ts^F/O driver and stained for EdU incorporation. Nuclei are stained with DAPI (blue). Red arrows point to pairs of EdU^+^ SC nuclei and yellow arrows point to pairs of EdU^-^ SC nuclei for each transgene (or in some cases, single nuclei when Rbf^CA^ is expressed). (I-N) Bar charts depicting mean % of EdU+ SCs per gland (I-K), the mean SC:MC nuclear area (L) and the geometric mean SC:MC nuclear area (M, N) of virgin males for control glands and glands expressing different transgenes in SCs. Dad cannot suppress E2F1/DP-mediated SC endoreplication and has only a limited effect on E2F1/DP-induced growth (B-D), but Dad is able to strongly suppress CycD/Cdk4-mediated SC endoreplication and growth (E). Rbf^CA^ suppresses most of the growth induced by Tkv^act^, however Rbf^CA^ is not able to suppress Tkv^act^-induced endoreplication(F-H). (O) Schematic showing interactions between BMP signalling (Tkv activation) and CycD/Rbf/E2F1-regulated endoreplication and cell growth. Note that BMP signalling activates SC endoreplication and associated growth via E2F1 in mated males, but for endoreplication-independent growth, it acts upstream of Rbf (new findings shown in red). Kruskal Wallis test; Dunn’s post hoc test (I-K). Welch ANOVA; Games-Howell post hoc test (L). Welch ANOVA on log-transformed data; Games-Howell post hoc test (M, N). n≥9 glands (I); n≥9 glands (J); n≥6 glands (K); n≥12 cells (L); n≥18 cells (M); n≥13 cells (N). Scale bars correspond to 20 μm. The error bars show the standard deviation (I-L) and geometric standard deviation (M, N) within the sample. 0.01<*p<0.05; 0.001<**p≤0.01; 0.0001<***p≤0.001; ****p≤0.0001.

Since BMP signalling also drives endoreplication and growth in SCs [2], we investigated whether the BMP signalling pathway interacts with the CycD/Rbf/E2F1 signalling axis in these cells. We first tested the effect of co-expressing E2F1/DP with a BMP antagonist, Daughters against Decapentaplegic (Dad) in SCs of virgin males. As discussed above, E2F1/DP overexpression stimulated endoreplication and growth (Fig 5A and 5C), while Dad overexpression suppresses SC growth in virgin males (Fig 5B). When co-expressed, all the SCs endoreplicated (Fig 5D and bar chart in Fig 5I). Furthermore, growth was much higher than in control glands and not significantly decreased from cells expressing E2F1/DP alone (Fig 5A–5D, bar chart in Fig 5L).

By contrast, when CycD/Cdk4 was co-expressed with Dad, Dad completely suppressed all SC growth induced by CycD/Cdk4 alone (compare Fig 5E with Fig 5A and 5B and S2I Fig, bar chart in Fig 5M). Additionally, only 20% of SCs showed any endoreplication (Fig 5E, bar chart in Fig 5J), despite the fact that CycD/Cdk4 can induce many rounds of endoreplication in the absence of Dad (Fig 4G’). This residual level of DNA synthesis seems most likely due to Dad expression incompletely suppressing BMP signalling in some SCs throughout the six-day expression period. Therefore, we conclude that CycD acts upstream of BMP signalling’s effects on endoreplication and growth; in turn, BMP signalling is upstream of E2F1/DP.

In order to further investigate how the BMP pathway interacts with the CycD/Rbf/E2F1 axis, we co-expressed Rbf^CA^ and a constitutively active form of a BMP Type-I receptor, Thick veins, (Tkv^act^) in SCs from virgin males. As previously reported, overexpression of Tkv^act^ induced endoreplication in most SCs of virgin males and high levels of growth (Fig 5A and 5G, bar charts in Fig 5K and 5N) [7], while overexpressing Rbf^CA^ alone suppressed SC growth (Fig 5B, bar charts in Fig 5K and 5N). Importantly, 78 ± 29% of SCs still endoreplicated when these two transgenes were co-expressed, phenocopying the effect of Tkv^act^ alone, even though the cells remained mononucleated (compare Fig 5H with 5F and 5G, bar chart in Fig 5K). By contrast, growth was similar to controls in virgin males (Fig 5A and 5F–5H, bar chart in Fig 5N); it was much reduced compared to when only Tkv^act^ was expressed and was significantly increased when compared to when Rbf^CA^ was expressed alone. Overall, we conclude that BMP signalling acts downstream of CycD/Rbf signalling to control endoreplication, because it has a dominant effect on this process when combined with CycD/Rbf modulators. By contrast, Rbf^CA^ overexpression suppresses the growth-promoting effects of BMP signalling on SC nuclei independently of endoreplication, suggesting that these two signals interact in different ways to control endoreplication-associated versus -independent growth (Fig 5O).

**Fig 6:**
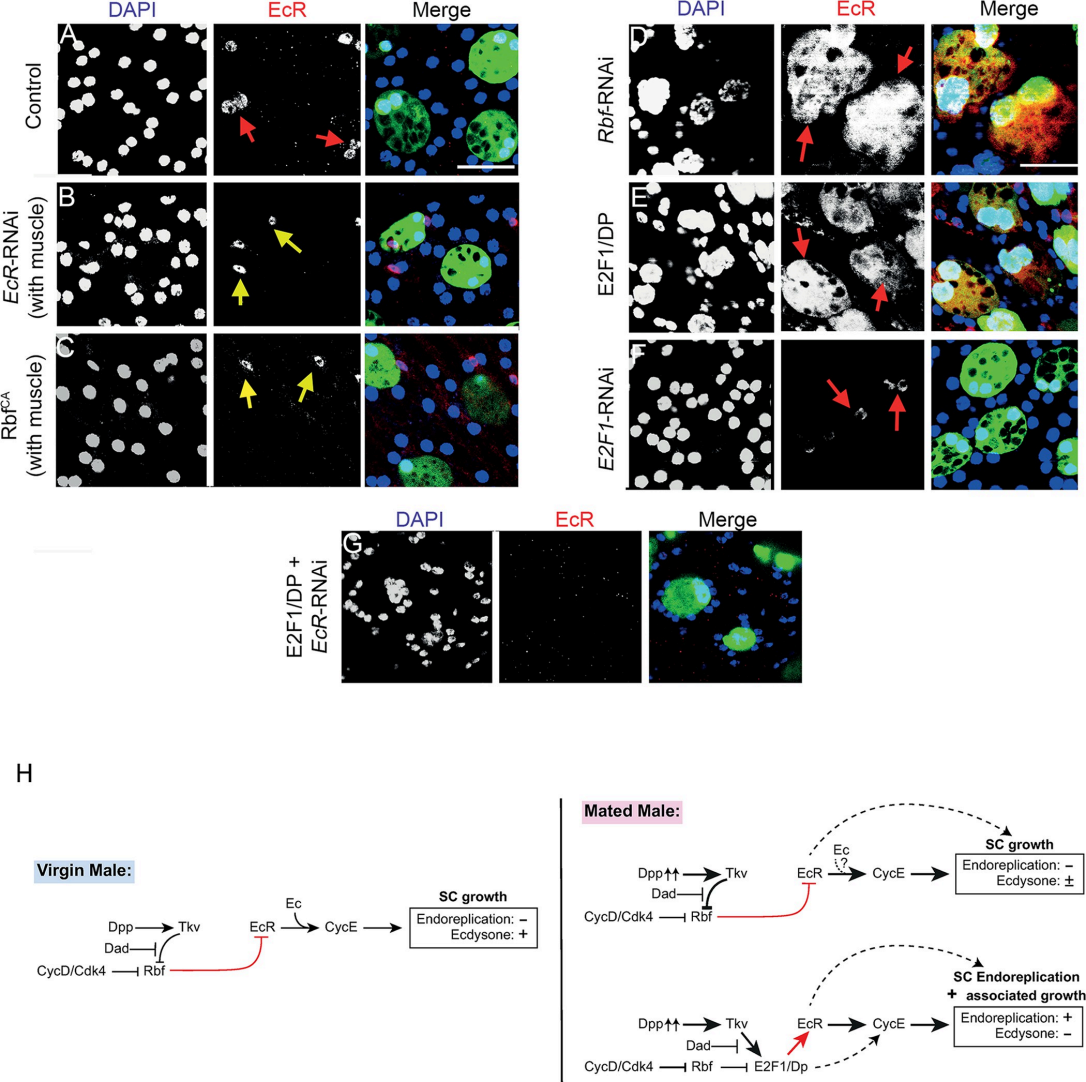
Rbf/E2F1 regulates EcR protein levels in SCs. (A-G) Images show distal tip of AGs from 6-day-old adult virgin males expressing nuclear GFP alone (control; this also stains the cytosol; A) or in combination with additional transgenes in SCs under the control of the esg^ts^F/O driver and stained with a pan-EcR antibody (red). Nuclei are stained with DAPI (blue). Red arrows mark SCs expressing EcR. Yellow arrows mark muscle cells expressing EcR as a control for EcR staining in glands that do not have EcR expression in SCs (B, C). Rbf negatively regulates EcR protein levels in SCs (C, D). Although overexpression of E2F1/DP results in an increase in EcR expression in SCs (E), which is completely suppressed by *EcR* knockdown (G), expression of *E2F1*-RNAi does not have a strong effect on EcR protein levels (F). (H) Schematic showing that for endoreplication-independent growth in mated males and for growth in virgin males, *CycD* and *Rbf* appear to control growth at least partly through the regulation of EcR levels via a mechanism that does not appear to involve *E2F1*. By contrast, endoreplication and associated growth are driven by CycD/Rbf/E2F1 signalling and also appear to involve increased EcR levels (new findings shown in red). Scale bars correspond to 50 μm.

Elevated BMP signalling is known to dramatically increase EcR protein expression in SCs, presumably contributing to the effects of this signalling pathway on SC growth and endoreplication [2]. Since E2F1 functions downstream of BMP signalling in endoreplication control, we hypothesised that Rbf/E2F1 might also affect EcR protein levels. Indeed, EcR protein, detected using a pan-EcR antibody, was greatly increased in comparison to control cells, when either E2F1/DP or *Rbf*-RNAi were overexpressed in SCs, with EcR present in the cytoplasm, as well as in all nuclei (compare Fig 6D and 6E to 6A). Expressing Rbf^CA^ in SCs completely suppressed EcR protein expression (Fig 6C), mirroring the phenotype observed with expression of *EcR*-RNAi (Fig 6B). However, *E2F1*-RNAi did not have a strong effect on EcR protein levels in SCs (Fig 6F), perhaps explaining the weaker effects of this knockdown on SC growth compared to Rbf^CA^ (Fig 4L and 4M). This is in sharp contrast to the effects on EcR levels, endoreplication and growth observed when E2F1/DP was overexpressed (Figs 4E and 6E). To confirm specific staining with the EcR antibody, we expressed E2F1/DP with *EcR*-RNAi in SCs; no EcR expression was observed (Fig 6G). We conclude that the BMP-regulated CycD/Rbf/E2F1 axis controls not only SC endoreplication and growth, but also expression of EcR protein, another key player in SC growth (Fig 6H). However, E2F1 does not play a critical role in normal endoreplication-independent control of EcR levels.

### Rbf and E2F1 function upstream of the EcR in the hormone-independent regulation of SC endoreplication

As discussed earlier, *Rb* loss and E2F1 activation increase AR levels in prostate cancer and are associated with CRPC, in which the AR can promote cell growth and proliferation in a hormone-independent fashion. We have previously shown that BMP signalling, which we have here demonstrated acts through E2F1 to control EcR-mediated endoreplication, switches on hormone-independent EcR activity [2]. Hence, we tested the functional interactions between the CycD/Rbf/E2F1 signalling axis and the EcR steroid receptor to determine whether this signalling drives ecdysone-independent, EcR-dependent endoreplication and associated growth in SCs, mirroring the effects in CRPC.

**Fig 7:**
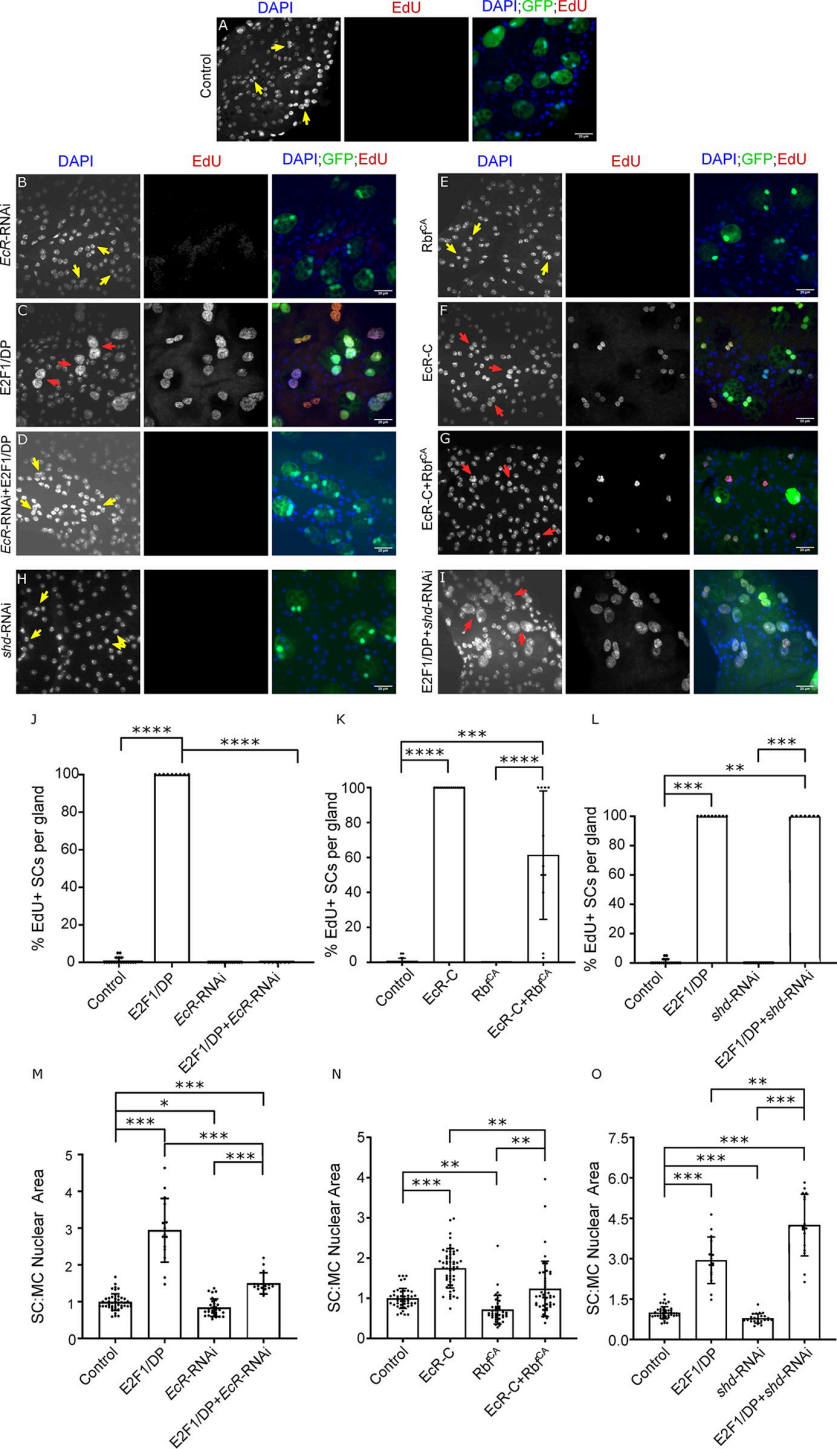
Rbf/E2F1 interacts with the EcR to regulate SC hormone-independent endoreplication and growth. (A-I) Images show distal tip of AGs from 6-day-old adult virgin males expressing nuclear GFP alone (control; this also stains the cytosol; A) or in combination with additional transgenes in SCs under the control of the esg^ts^F/O driver and stained for EdU incorporation. Nuclei are stained with DAPI (blue). Red arrows point to pairs of EdU^+^ SC nuclei and yellow arrows point to pairs of EdU^-^ SC nuclei for each transgene (or in some cases, single nuclei when Rbf^CA^ is expressed). (J-O) Bar charts depicting mean % of EdU+ SCs per gland (J-L), the geometric mean SC:MC nuclear area (M, N), mean SC:MC nuclear area (O) of virgin males for control glands and glands expressing different transgenes in SCs. E2F1-mediated endoreplication in SCs requires ecdysone-independent EcR signalling (C, D, H-J, L). However, E2F1 can promote some growth in SCs independently of EcR (D, I, M, O). Rbf and EcR may partially act independently of each other, at least to regulate SC growth (E-G, K, N). E2F1’s effects on growth and endoreplication do not appear to require ecdysone, which is required for normal SC growth in virgin males (H, I, L, O). Kruskal Wallis test; Dunn’s post hoc test (J-L). Welch ANOVA on log-transformed data; Games-Howell post hoc test (M-N). Welch ANOVA; Games-Howell post hoc test (O). n≥7 glands (J); n≥11 glands (K); n≥7 glands (L); n≥15 cells (M); n≥39 cells (N); n≥15 cells (O). Scale bars correspond to 20 μm. The error bars show the standard deviation (J-L, O) and the geometric standard deviation (M-N) within the sample. 0.01<*p<0.05; 0.001<**p≤0.01; 0.0001<***p≤0.001; ****p≤0.0001.

When E2F1/DP was co-expressed with *EcR*-RNAi in SCs of virgin males, the endoreplication induced when E2F1/DP is expressed alone (Fig 7C) was completely suppressed (compare Fig 7D with Fig 7A–7C, bar chart in Fig 7J). SC nuclei grew to a greater size than controls or SCs expressing *EcR*-RNAi, but growth was very strongly suppressed compared to cells expressing E2F1/DP alone (Fig 7A–7D and 7M). Hence, we conclude that E2F1/DP can elicit some SC growth when EcR expression is suppressed (EcR expression levels were undetectable under these conditions; Fig 6G), but, perhaps surprisingly, for SC endoreplication, E2F1/DP requires the EcR to drive this CycE-dependent event.

Co-expressing Rbf^CA^, which blocks SC endoreplication in mated males (Fig 4D), with endoreplication- and growth-inducing EcR-C in SCs of virgin males produced more variable and intermediate phenotypes, probably because each SC expresses different relative levels of these transgenes (compare Fig 7G with Fig 7E and 7F, bar charts in Fig 7K and 7N). Whereas 100% of SCs expressing EcR-C alone endoreplicated in virgin males, only 60% endoreplicated when Rbf^CA^ was co-expressed (Fig 7K), with some glands exhibiting endoreplication in 100% of SCs and others in only 2.5%. No endoreplication was observed in SCs expressing Rbf^CA^ alone (Fig 7E and 7K). These results suggest that EcR-C is able to induce endoreplication even when SCs express Rbf^CA^, consistent with our finding that EcR acts downstream of E2F1 in endoreplication control. Furthermore, growth levels in SCs of these males were similar to controls (Fig 7N), an intermediate phenotype that suggests Rbf can act somewhat independently of EcR, presumably to control endoreplication-associated growth, mirroring our findings when E2F1/DP is overexpressed (Figs 6H and 7M).

Finally, since endoreplication is regulated by the EcR in a hormone-independent fashion, we hypothesised that E2F1 activation should also stimulate EcR-mediated genome replication in the absence of hormone, mirroring events in CRPC. Even though, as we have previously shown [2], expression of an RNAi targeting *shd*, the gene encoding ecdysone 20-hydroxylase required for the last step in 20-hydroxyecdysone synthesis, suppressed hormone-dependent SC growth in virgin males (Fig 7H and 7O), co-expression with E2F1/DP did not suppress the endoreplication that this transcriptional complex induced (compare Fig 7I with Fig 7C, bar chart in Fig 7L). In fact, co-expression of E2F1/DP with *shd*-RNAi resulted in an increase in growth compared to E2F1/DP expression alone (Fig 7I, bar chart in Fig 7O), suggesting ecdysone may interfere with some aspects of E2F1/DP-induced growth. This is in sharp contrast to E2F1/DP co-expression with EcR-RNAi, which suppresses all endoreplication and most E2F1/DP-induced growth (compare Fig 7D with 7C, bar chart in Fig 7J and 7M). Overall, we conclude that E2F1/DP-induced SC endoreplication is mediated by hormone-independent EcR signalling.

## Discussion

**Fig 8:**
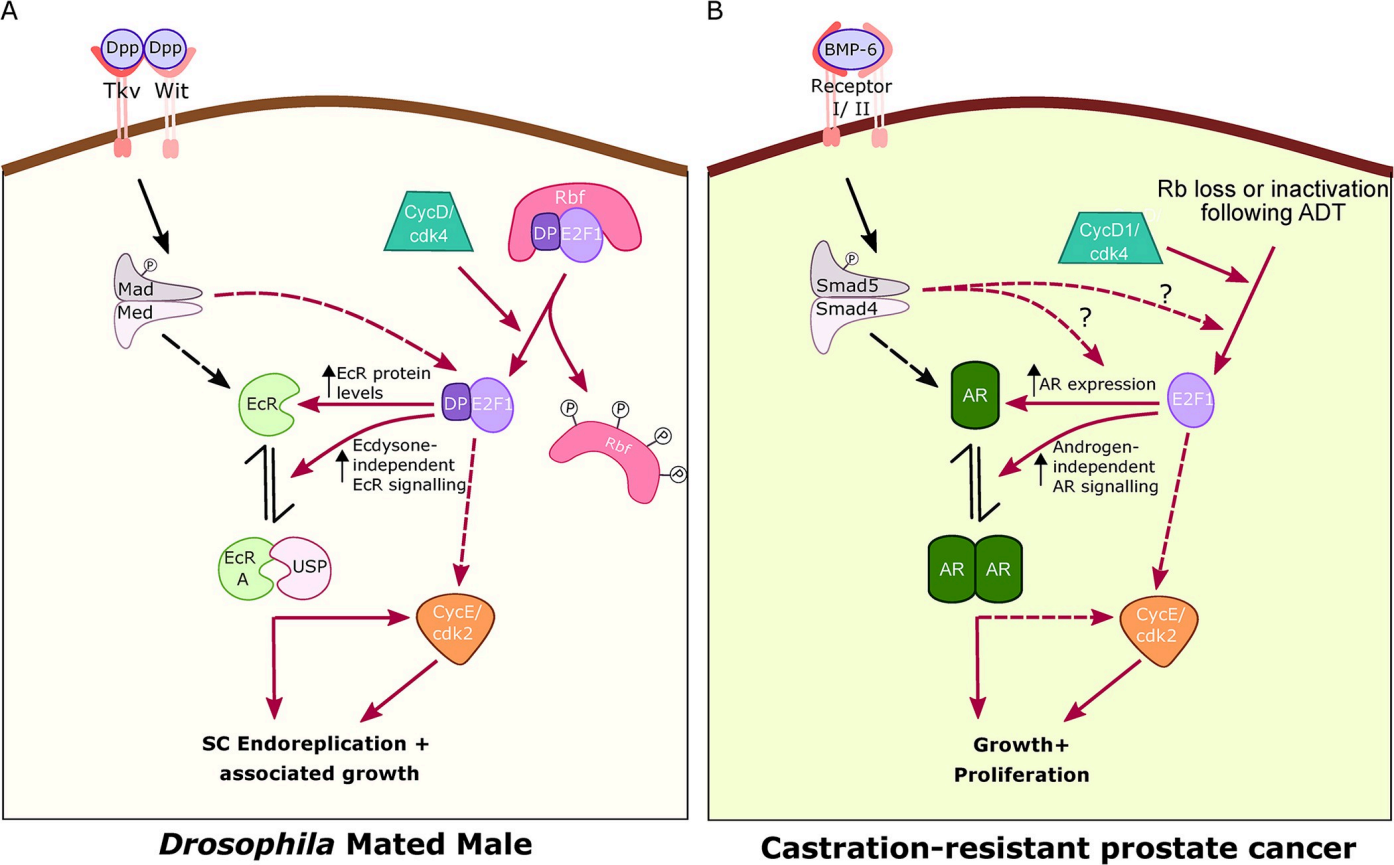
Regulation of SC genome endoreplication by cell cycle regulators parallels control of tumour cell growth in CRPC. (A) CycD/Rbf/E2F1/CycE are all essential for the endoreplication in SCs that is induced by mating. Hormone-independent EcR signalling appears to be required for E2F1-dependent induction of endoreplication and associated growth, an effect that is at least partly mediated by increasing EcR levels. In turn, the EcR, in a complex with its partner Usp ^2^, is at least partly dependent on CycE to perform its functions in endoreplication. BMP signalling interacts with the cell cycle regulatory axis downstream of Rbf, but upstream of E2F1, although our data do not eliminate the possibility that it may also act on the EcR in an E2F1-independent fashion to control endoreplication-independent growth. The signalling network that controls endoreplication and associated growth has not been identified in other *Drosophila* cells to date. (B) However, mirroring this regulation in SCs, activation of CycD/Rbf/E2F1 during ADT, leads to an increase in AR expression and hormone-independent AR signalling that drive growth and proliferation in CRPC. BMP-6 signalling may also be involved in this process. Therefore, in SCs after mating and in CRPC, the cell cycle machinery performs a distinct role involving elevated steroid receptor expression and hormone-independent, steroid receptor-dependent signalling to drive DNA replication and growth. Cdk4/2- cyclin dependent kinase 4/2; CycD- Cyclin D; CycE- Cyclin E; Dp- Dumpy; Dpp- Decapentaplegic; Ec-Ecdysone; EcR- Ecdysone receptor; Med- Medea (*Drosophila* co-Smad); P- Phosphate group; Rbf- Retinoblastoma; Tkv- Thickveins (type I BMP receptor); Usp- Ultraspiracle (EcR binding partner); Wit- Wishful thinking (type II BMP receptor); AR- Androgen Receptor; Smad5- human receptor Smad; Smad4- human co-Smad. Red arrows depict the pathways that regulate SC endoreplication and associated growth, and the dotted arrows show probable signalling interactions.

We have previously shown that endoreplication and growth (assessed by measuring nuclear growth) in *Drosophila* SCs requires the EcR and that this receptor functions in a hormone-independent way after mating to activate endoreplication and associated growth [2]. This mechanism may permit SCs to secrete rapidly and contribute to the refilling of the AG lumen after mating, even if levels of circulating 20-hydroxyecdysone in the male are low. Here, informed by studies of CRPC, where late-stage *Rb* loss increases AR levels and activates hormone-independent AR signalling, we investigated how *Drosophila* Rbf and other interacting cell cycle regulators impact hormone-independent EcR signalling, endoreplication and associated growth in SCs. We found that Rbf inhibits hormone-independent endoreplication observed after mating. Remarkably, activation of the CycD/Rbf/E2F1 axis requires the EcR for induction of SC CycE-dependent endoreplication (summarised in S1 Table). This physiological mechanism has not been reported in other *Drosophila* cell types, but does mirror changes seen in CRPC (Fig 8), suggesting the possibility that the latter pathological change might reflect defective signalling involving a physiological regulatory network in human prostate epithelium that is yet to be characterised.

### Activated Rbf reverses the binucleate state in adult SCs independently of its effects on growth and endoreplication

Our study revealed an interesting additional role for Rbf in controlling the nucleation state in binucleate adult SCs. Rbf^CA^ reversibly induces almost all SCs to become mononucleate (Fig 2C), but has no effect on MC nucleation state (Fig 2D). Furthermore, knocking down *E2F1* in SCs results in just a few SCs becoming mononucleate (S1A Fig), whereas no effect was observed when *E2F1* was knocked down in MCs (S1B Fig). Therefore, SCs appear to require some signalling by the CycD/Rbf/E2F1 axis to retain their binucleate state in adults. Currently, it is unclear whether SCs become mononucleate because of nuclear fusion or nuclear loss. We have not seen convincing evidence for either event to date, which may suggest that the change is relatively rapid and might therefore only be observed with extended time-lapse imaging.

Perhaps most surprisingly, expression of the Rbf^CA^ construct in MCs results in SC mononucleation (Fig 2D), while MCs retain their binucleate nature. In this case, those SCs, which become mononucleate, are still able to endoreplicate upon mating. Hence, it appears that an unknown secreted MC signal, which is not Rbf, can direct SC mononucleation, or this signal promotes binucleation in SCs and is suppressed by Rbf^CA^. Since MCs do not become mononucleated, MCs either must lack the machinery to respond to or modulate this signal, or regulate binucleation in a different way. *E2F1*-RNAi expression in MCs does not have the capacity to elicit the mononucleation signal to SCs, indicating that E2F1 is not involved in this signalling mechanism, although this might alternatively be accounted for by insufficient knockdown of *E2F1* in MCs. Interestingly, a recent study has shown that although Rb/E2F does not initiate binucleation in mammalian cardiomyocytes, the binucleate and mononucleate cells have distinct Rb/E2F-mediated transcriptional programmes [23], thereby emphasising the importance of this pathway in different nucleation states.

### The CycD/Rbf/E2F1 signalling axis and CycE regulate the transition from hormone-dependent to hormone-independent EcR signalling in SCs

We have demonstrated that CycD and CycE, are necessary and sufficient for the induction of endoreplication in SCs (see models in Figs 6H and 8A), and that they are also required for ecdysone-dependent SC growth in virgin males, even though no detectable DNA replication occurs in these cells (Fig 6H). However, they are not needed for at least some of the endoreplication-independent SC growth that occurs in mated males. In breast cancer and prostate cancer, CycD1 and CycE have been demonstrated to interact with and regulate both ligand-dependent and ligand-independent steroid receptor signalling, the latter signalling playing a vital role in the initiation of the hormone-refractory form of cancer growth [24–26].

CycD-regulated Rbf is also involved in normally suppressing these SC growth regulatory functions, although unlike with *CycD* knockdown, overexpression of a constitutively active form of Rbf, which may induce effects that are not observed under any normal physiological conditions, appears to be able to block all growth, even in mated males. By contrast, although E2F1 is necessary and sufficient to promote endoreplication in SCs, neither it nor E2F2 are essential for growth that occurs in virgins. In fact, E2F1 appears to inhibit endoreplication-independent growth in mated males, suggesting that Rbf and E2F1 can act independently to fulfil some of their growth-regulatory activities in SCs (Fig 6H). Rb functions that occur independently of its effects on E2F transcriptional activity have been observed in Rb-mediated cell cycle arrest and chromatin stability in mammalian cells [27]. Furthermore, the E2F1-independent Rb activity that regulates chromatin condensation appears to be conserved in *Drosophila* [28]. There is also some evidence suggesting E2F1 activity may not be affected by Rbf during early fly embryogenesis [29]. However, even if these two proteins can act independently in some contexts in SCs, canonical Rbf/E2F1 signalling is also active in these cells, and is required for endoreplication-associated SC growth in mated males (Fig 6H).

Rbf and E2F1 can mediate their effects on SC endoreplication and growth partly by regulating EcR protein levels, as previously observed for BMP/Dpp signalling [2]. Epistasis experiments suggest that the BMP and EcR pathways interact with the CycD/Rbf/E2F1/CycE axis in SCs at different points. BMP signalling intersects the cell cycle regulatory pathway upstream of E2F1 activity and CycE, and downstream of CycD. It also lies downstream of Rbf for SC endoreplication and associated growth (Fig 6H). EcR lies downstream of CycD/Rbf/E2F1, but upstream of CycE in the regulation of endoreplication and growth in SCs, although our *CycE* knockdown experiments, when an activated form of EcR is overexpressed (Fig 3G and 3J), suggest that not all EcR-mediated endoreplication may require CycE. However, an alternative interpretation of these results is that in the presence of the UAS-*EcR* transgene, which drives expression of EcR at non-physiological levels, UAS-driven *CycE* knockdown is reduced and therefore does not completely suppress all CycE-dependent effects, as it does in the absence of UAS-*EcR*. We cannot easily eliminate this explanation by qRT-PCR or western blot analysis of AG tissue, because SCs make up such a small proportion (< 4%) of AG cells.

Importantly, we conclude that E2F1 induces endoreplication and growth in an ecdysone-independent fashion, which mirrors the effects observed with loss of Rb and unsupervised activation of E2F activity in CRPC (Fig 8). Similar to SCs, BMP signalling has also been implicated in the emergence of CRPC [11], where signalling by BMP-6 drives increased AR expression and castration-resistance.

SCs contain approximately ten highly enlarged secretory compartments [4]. About four of these are released from each SC during mating and roughly one third of the luminal content of the AG is transferred to females. Therefore, rapid replenishment and secretion of SC secretory compartments after mating is essential to maintain male fecundity. Why might this EcR-mediated process be hormone-independent? Whole-animal 20-HE titres increase in male flies exposed to previously mated females [8]. Furthermore, normal courtship behaviours are dependent on EcR signalling in the brain [8,30]. Organisms may often reduce levels of steroids that control sexual behaviour and reproductive activity to spare resources, either prior to reaching full sexual maturity or in the absence of potential mates. Circulating steroid levels and associated signalling outputs generally change slowly. Therefore, rapid changes in steroid receptor-mediated responses, as seen in SCs, can only be achieved through hormone-independent mechanisms. Recruiting the CycD/Rbf/E2F1 pathway, a key regulator of cell growth, proliferation and endoreplication, to control the switch to hormone-independent EcR signalling, couples this switch to well-established cell biological events that stimulate secretion [22]. Furthermore, by controlling the activity of this pathway via BMP signalling, activation is directly linked to the elevated autocrine BMP signalling that takes place during mating [4].

### The switch to hormone-independent EcR signalling mirrors CRPC signalling

We have shown that in *Drosophila* SCs, which share several cell biological similarities with prostate epithelial cells, the fly *Rb* homologue, *Rbf*, suppresses ecdysone-independent EcR-mediated growth, which is induced after male flies mate. To our knowledge, such regulation of EcR signalling has not previously been observed in flies. However, the signalling network that controls endoreplication in SCs shares many similarities with the signalling observed in castration-resistant prostate cancer (Fig 8). Multiple mechanisms can contribute to the inevitable emergence of CRPC following androgen-deprivation therapy, including the late-stage loss of *Rb*, a change that occurs at much earlier stages in most other cancers. In the prostate, this genetic change increases AR levels and hormone-independent AR signalling [10], mirroring the effects on EcR in SCs.

Comprehensive genomic profiling of prostate tumours, cell lines and xenografts has revealed that Rb signalling is functionally reduced in 34% of primary tumours and in 74% of castration-resistant tumours, thereby stressing the importance of Rb loss in the emergence of CRPC [31]. Studies in prostate cancer cell lines have demonstrated that Rb/E2F1 interacts with [12] and stimulates *AR* transcription, AR protein expression and AR target gene expression [10,32] to facilitate the development of hormone-refractory prostate cancer. Interestingly, in a *Drosophila* spinal and bulbar muscular atrophy model, it was observed that the *Drosophila* Rbf/E2F1 axis is sufficiently conserved in evolution to functionally interact with human polyglutamine repeat expansion AR mutants to mediate neurodegeneration in *Drosophila* eyes [33,34]. In fact, the link between *Rb* loss and resistance to steroid hormone deprivation therapy may extend beyond prostate cancer with *Rb* mutations found to be associated with resistance to estrogen deprivation in estrogen receptor-positive breast cancer [35,36]. However, in this case, *Rb* loss is not reported as a late-stage event in breast cancer progression.

The endoreplication-independent regulation of *Drosophila* SC growth by BMP signalling, CycD/Rbf and EcR is unusual in that it appears to lack a contribution from E2F transcription factors and is also modulated by mating state. By contrast, the regulation of endoreplication and endoreplication-associated growth is absolutely dependent on the well-established CycD/Rbf/E2F1/CycE cascade, but also has a key requirement for hormone-independent EcR activation (see also [2]). Although we have not been able to assess the highly cell type-specific physical interaction between EcR and Rbf/E2F1, because of the small number of SCs in each male, it seems likely that this part of the Rbf/E2F1-dependent regulation of EcR that controls its hormone-independent signalling involves physical interaction between the EcR and at least E2F1, as reported in prostate cancer cells.

Why would the physiological control of SC endoreplication in mated male flies share regulatory parallels with the hormone-independent, AR-induced growth and proliferation observed in pathological CRPC (Fig 8)? One obvious explanation is that the aberrant signalling observed in CRPC represents the dysregulation of an unidentified physiological control mechanism in human prostate cells that permits functional AR signalling when androgen levels are low, for example during reproductive maturation. Whether or not this is the case, our findings indicate that SC endoreplication could be used as a model to further investigate some of the additional genetic changes, which, like the blockade of BMP signalling, might suppress the emergence of CRPC in future therapies.

## Materials and methods

### *Drosophila* Stocks and fly husbandry

The following fly stocks were obtained from the Bloomington *Drosophila* Stock Center, unless other source is mentioned: esg^ts^F/O (*w*^*-*^*; esg-GAL4*, *UAS-GFP*_*nls*_*; act>CD2>GAL4*, *UAS-FLP*; gift from B. Edgar) [37], *UAS-EcR-RNAi* (TriP.JF02538) [38], *UAS-EcR-C* [39], *UAS-CycE-RNAi* (TriP.GL00511), *UAS-CycE* [40], *UAS-Rbf*^*CA*^ (w[*]; P{w[+mC] = UAS-Rbf.280}3/TM3, Sb[1]) [19], *UAS-Mud-RNAi* (TriP.HMS01458) [41], *UAS-CycD/cdk4* (gift from B.Edgar) [42], *UAS-CycD-RNAi* (TriP.HMS00059) [43], *UAS-Rbf-RNAi* (TriP.HMS03004) [44], *UAS-E2F1/Dp*, *UAS-E2F1-RNAi* (TriP. JF02718) [45], *UAS-Tkv*^*act*^ (gift from K. Basler) [46], *UAS-Dad* (gift from D. Bennett) [47], *UAS-shd-RNAi* [2]. Flies were reared in standard cornmeal agar medium and experimental crosses were maintained either at 18°C or 25°C. For endoreplication experiments, newly eclosed virgin males of the required genotype were collected and kept on food with or without 0.2 mM EdU (see below). For multiply mated experiments, each male was placed in individual vials with ten virgin *w*^*1118*^ females [2,4,7].

### Immunohistochemistry and microscopy

The protocol used for fixing and immunostaining the AG was performed as reported previously [2,4,7,21]. Fixed samples were blocked with PBSTG (PBST with 10% Goat Serum) for 30 min at room temperature and were incubated overnight with primary antibody diluted in PBSTG at 4°C. They were washed in PBST for 6 X 10 min before being incubated with secondary antibody (1:400 in PBST) (ThermoFisher) for 30 min at room temperature. Primary antibodies, anti-FAS3 (Developmental Biology Hybridoma Bank (DSHB); 1:10) and anti-EcR (DSHB; 1:10) were used in conjunction with fluorescent Alexa-555- (ThermoFisher; 1:400) and Cy3- (Jackson Laboratories; 1:400) conjugated donkey anti-mouse secondary antibody respectively. The glands were imaged using an upright Zeiss LSM 880 Airy Scan confocal microscope with the Zeiss Plan-Apochromat 63X/1.4 NA Oil objective (Carl Zeiss) and Zeiss Plan-Apochromat 40X/1.3 NA Oil objective (Carl Zeiss). Immersion oil of refractive index 1.514 (Cargill labs) was used.

### SC nuclear growth assay

Z-stack images of the whole SC (3 per gland) were acquired by confocal microscopy as discussed above. The sum of all stacked images was obtained and analysed using Fiji. Three of the most strongly expressing GFP-positive SCs were selected and the sum of the maximum areas of both nuclei and of an MC adjacent to each SC was calculated. In the case of mononucleate cells (e.g. Rbf^CA^), the area of the single nucleus was calculated. SC Nuclear Area/MC Nuclear Area was used as a measure of SC growth [2,7].

### EdU incorporation assay

The Click-iT Plus EdU Cell Proliferation Kit for Imaging (Invitrogen; Alexa Fluor 594 dye) was used to detect DNA replication. EdU is a synthetic analogue of thymidine. Flies were maintained on medium prepared by mixing standard yeast-cornmeal agar medium at 60°C with an 80 mM EdU (Cambridge Bioscience) stock solution (diluted in PBS, as per the manufacturer’s instructions) to reach a final EdU concentration of 0.2 mM. In order to detect EdU incorporation, Ags were dissected from flies fed on EdU-containing food, but the testes were kept attached as a positive control for EdU staining, because the adult stem cell niches in the testes undergo DNA replication and incorporate EdU [48]. Once suspended in PBST, tissues were transferred to PBSTG and incubated for 45 minutes at RT to block, before being washed with PBST. DNA was labelled by adding the Click-iT reaction mix (prepared following manufacturer’s instructions) to the vials and left to incubate for 30 min at room temperature, away from light. Glands were washed in PBST for 3 x 10 min and then re-suspended in PBS. Only AGs from flies whose testes were positive for EdU staining were included for the analysis. The total number of SCs were quantified by counting the number of GFP-containing SCs (nGFP from esg^ts^F/O driver). Percentage of SCs which incorporated EdU was then used as a measure for endoreplication in the gland.

### Statistical analyses

Either the mean or geometric mean of SC:MC nuclear area or mean rank of % of EdU incorporated were compared across different genotypes. The normality or log-normality of the data were checked using the Shapiro-Wilk normality test and D’Augostino and Pearsons’ normality test. For log-normal data, further analyses were undertaken using log-transformed data. Bartlett’s homogeneity of variance test was used to check for similarity of variances; if variances were similar, one-way ANOVA followed by Tukey’s HSD post-hoc test was used and if variances were dissimilar, Welch ANOVA followed by Games-Howell post-hoc test was used. If the data were neither normally nor log-normally distributed, Kruskal-Wallis test followed by Dunn’s post-hoc test was used. When the data are log-transformed, the analyses conducted compare the geometric means of the data rather than the arithmetic means.


$$\frac{\sum_{i=1}^{N}log(i)}{N}=log(\sqrt[{N}]{\prod_{i=0}^{N}i})$$

Where $\frac{\sum_{i=1}^{N}log(i)}{N}$ is the arithmetic mean of the log-transformed values and $\sqrt[{N}]{\prod_{i=0}^{N}i}$ is the geometric mean of the actual data.

Therefore, when log-normal data are represented in a scatterplot graph, we show the geometric means and the corresponding geometric standard deviations.

## Supporting information

S1 Fig E2F1 cell autonomously regulates SC nucleation state. (A,B) Images show distal tip of AGs from 6-day-old adult virgin males of glands expressing *E2F1*-RNAi under the control of the esg^ts^F/O driver (A) and under the control of the *Acp26Aa*-GAL4 driver (B), which drives nuclear GFP production (stains cytoplasm in A; see Fig 2H, and 2I for analysis). Dashed red ellipses mark the outlines of mononucleate SCs; dashed yellow ellipses mark the outlines of binucleate SCs. Scale bars correspond to 50 μm.
(DOCX)

S2 Fig Role of CycE and CycD in SC endoreplication and growth regulation. (A-D) Images show distal tip of AGs from 6-day-old adult virgin (A, C) or multiply mated (B, D) males expressing nuclear GFP alone (control; this also stains the cytosol, A, B) or in combination with *cycE*-RNAi (C, D) in SCs under the control of the esg^ts^F/O driver and stained for EdU incorporation in SCs. Nuclei are stained with DAPI (blue). Red arrows point to EdU^+^ SC nuclei and yellow arrows point to EdU^-^ SC nuclei for each transgene. (E, F) Bar charts depicting the mean % of EdU^+^ SCs per gland (E) and mean ratio of the size of SC nuclei relative to neighbouring MC nuclei (F) of virgin and mated flies expressing only nuclear GFP or also *CycE*-RNAi in SCs. Knocking down *CycE* completely inhibits SC endoreplication, but does not completely suppress SC growth that occurs after mating (C, D). (G-J) Images show distal tip of AGs from 6-day old virgin (G) and mated (H) adult males expressing nuclear GFP alone (control; this also stains the cytosol) or with either the combination of CycD/Cdk4 in SCs of virgin males (I) or CycD-RNAi (J) in SCs of mated males under the control of the esg^ts^F/O driver. (G’-J’) Images show lower magnification views of different EdU-stained AGs of same genotypes. AGs are outlined with dashed white lines and are labelled as AG. In J’, part of the ejaculatory duct is seen and has been labelled as ED. Kruskal Wallis test; Dunn’s post hoc test (E). Welch ANOVA; Games-Howell post hoc test (F). n≥9 glands (E); n≥15 cells (F). Scale bars correspond to 20 μm (A-D, G-J) and 50 μm (G’-J’). The error bars show the standard deviation within the sample. 0.01<*p<0.05; 0.001<**p≤0.01; 0.0001<***p≤0.001; ****p≤0.0001.
(TIF)

S3 Fig Validation of SC nuclear growth assay for glands containing mononucleate and binucleate cells. Bar chart depicting the mean cellular area of SCs from control glands or glands expressing Rbf^CA^ in SCs. The cellular area of SCs expressing Rbf^CA^ is smaller than SCs from control glands, mirroring the reduction observed with the SC nuclear area assay (see Fig 4L). Mann-Whitney test; n≥43 cells; ****p<0.0001.
(DOCX)

S4 Fig E2F2 does not appear to regulate SC endoreplication and growth. (A-D) Images show distal tip of AGs from 6-day old adult virgin (A, C) or multiply mated (B, D) males expressing nuclear GFP alone (control; this also stains the cytosol; A, B) or in combination with *E2F2*-RNAi (C, D) in SCs under the control of the esg^ts^F/O driver and stained for EdU incorporation in SCs. Nuclei are stained with DAPI (blue). Red arrows point to EdU^+^ pairs of SC nuclei and yellow arrows point to EdU^-^ pairs of SC nuclei for each transgene. (E, F) Bar charts depicting the geometric mean ratio of the size of SC nuclei relative to neighbouring MC nuclei (E) and mean % of EdU+ SCs per gland (F) in virgin and mated flies expressing no other transgene or *E2F2*-RNAi in SCs. Knocking down *E2F2* does not significantly affect SC growth or endoreplication in either virgin or mated males. One-way ANOVA on log-transformed data; Tukey’s HSD post-hoc test (E). Kruskal-Wallis test; Dunn’s post-hoc test (F). n≥18 (E); n≥6 (F). 0.0001<***p≤0.001.
(DOCX)

S1 Table Summary of genes and genetic interactions regulating SC endoreplication and growth. Table summarises the different genetic manipulations used in this study, the signalling pathway or gene that is tested by these manipulations, and the effects on SC endoreplication and nuclear growth (used as a proxy for SC growth) in virgin and mated males. Note that since endoreplication does not normally occur in virgin males, manipulations, which reduce endoreplication, will have no effect in virgins. In some genetic backgrounds, mating males no longer affects SC growth relative to virgin males (marked ‘No’), unlike in control males and other backgrounds, where SCs from multiply mated males grow more than SCs from virgins (marked ‘Yes’). On the right hand side, a summary of the epistatic interactions between different genes is provided. Note that the BMP signalling pathway affects endoreplication-dependent and endoreplication-independent growth via different routes (E2F1-dependent and -independent respectively), and that it remains unclear whether CycE mediates all the mating-dependent effects of the EcR on endoreplication and growth (see Fig 6H).
(XLSX)

S1 Data Files Datasets for Figures and Supplementary Information. (XLSX)
