# Supplementary figures and images for: Rbf/E2F1 control growth and endoreplication via steroid-independent Ecdysone Receptor signalling in Drosophila prostate-like secondary cells
Source: PLoS Genet. 2023 Jun 26;19(6):e1010815. doi: 10.1371/journal.pgen.1010815 (PMC10328346; doi:10.1371/journal.pgen.1010815)

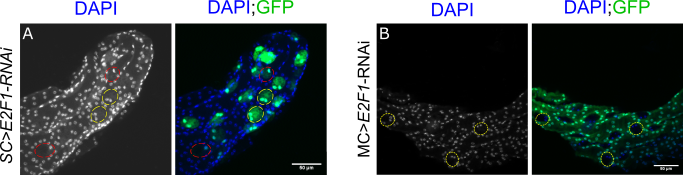
**Figure S1:**

Supplement: S1 Fig — (A,B) Images show distal tip of AGs from 6-day-old adult virgin males of glands expressing E2F1-RNAi under the control of the esgtsF/O driver (A) and under the control of the Acp26Aa-GAL4 driver (B), which drives nuclear GFP production (stains cytoplasm in A; see Fig 2H, and 2I for analysis). Dashed red ellipses mark the outlines of mononucleate SCs; dashed yellow ellipses mark the outlines of binucleate SCs. Scale bars correspond to 50 μm. (DOCX) [file pgen.1010815.s001.docx]

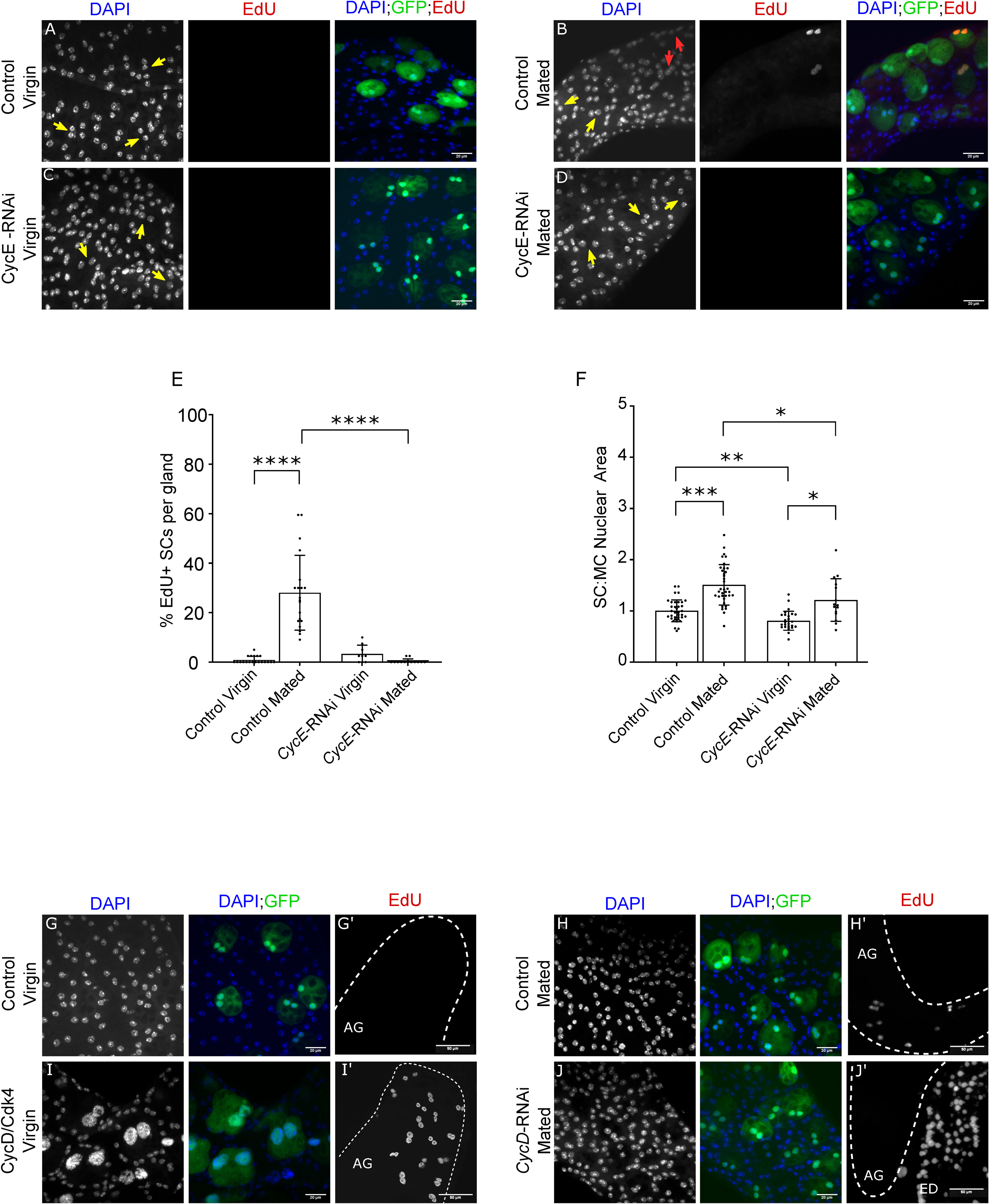

Supplement: S2 Fig — (A-D) Images show distal tip of AGs from 6-day-old adult virgin (A, C) or multiply mated (B, D) males expressing nuclear GFP alone (control; this also stains the cytosol, A, B) or in combination with cycE-RNAi (C, D) in SCs under the control of the esgtsF/O driver and stained for EdU incorporation in SCs. Nuclei are stained with DAPI (blue). Red arrows point to EdU+ SC nuclei and yellow arrows point to EdU- SC nuclei for each transgene. (E, F) Bar charts depicting the mean % of EdU+ SCs per gland (E) and mean ratio of the size of SC nuclei relative to neighbouring MC nuclei (F) of virgin and mated flies expressing only nuclear GFP or also CycE-RNAi in SCs. Knocking down CycE completely inhibits SC endoreplication, but does not completely suppress SC growth that occurs after mating (C, D). (G-J) Images show distal tip of AGs from 6-day old virgin (G) and mated (H) adult males expressing nuclear GFP alone (control; this also stains the cytosol) or with either the combination of CycD/Cdk4 in SCs of virgin males (I) or CycD-RNAi (J) in SCs of mated males under the control of the esgtsF/O driver. (G’-J’) Images show lower magnification views of different EdU-stained AGs of same genotypes. AGs are outlined with dashed white lines and are labelled as AG. In J’, part of the ejaculatory duct is seen and has been labelled as ED. Kruskal Wallis test; Dunn’s post hoc test (E). Welch ANOVA; Games-Howell post hoc test (F). n≥9 glands (E); n≥15 cells (F). Scale bars correspond to 20 μm (A-D, G-J) and 50 μm (G’-J’). The error bars show the standard deviation within the sample. 0.01<*p<0.05; 0.001<**p≤0.01; 0.0001<***p≤0.001; ****p≤0.0001. (TIF) [file pgen.1010815.s002.tif]

**Figure S3:**

**
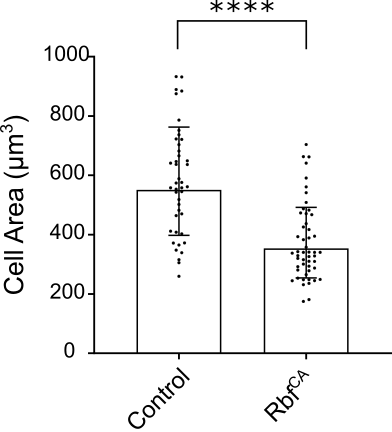
**

Supplement: S3 Fig — Bar chart depicting the mean cellular area of SCs from control glands or glands expressing RbfCA in SCs. The cellular area of SCs expressing RbfCA is smaller than SCs from control glands, mirroring the reduction observed with the SC nuclear area assay (see Fig 4L). Mann-Whitney test; n≥43 cells; ****p<0.0001. (DOCX) [file pgen.1010815.s003.docx]

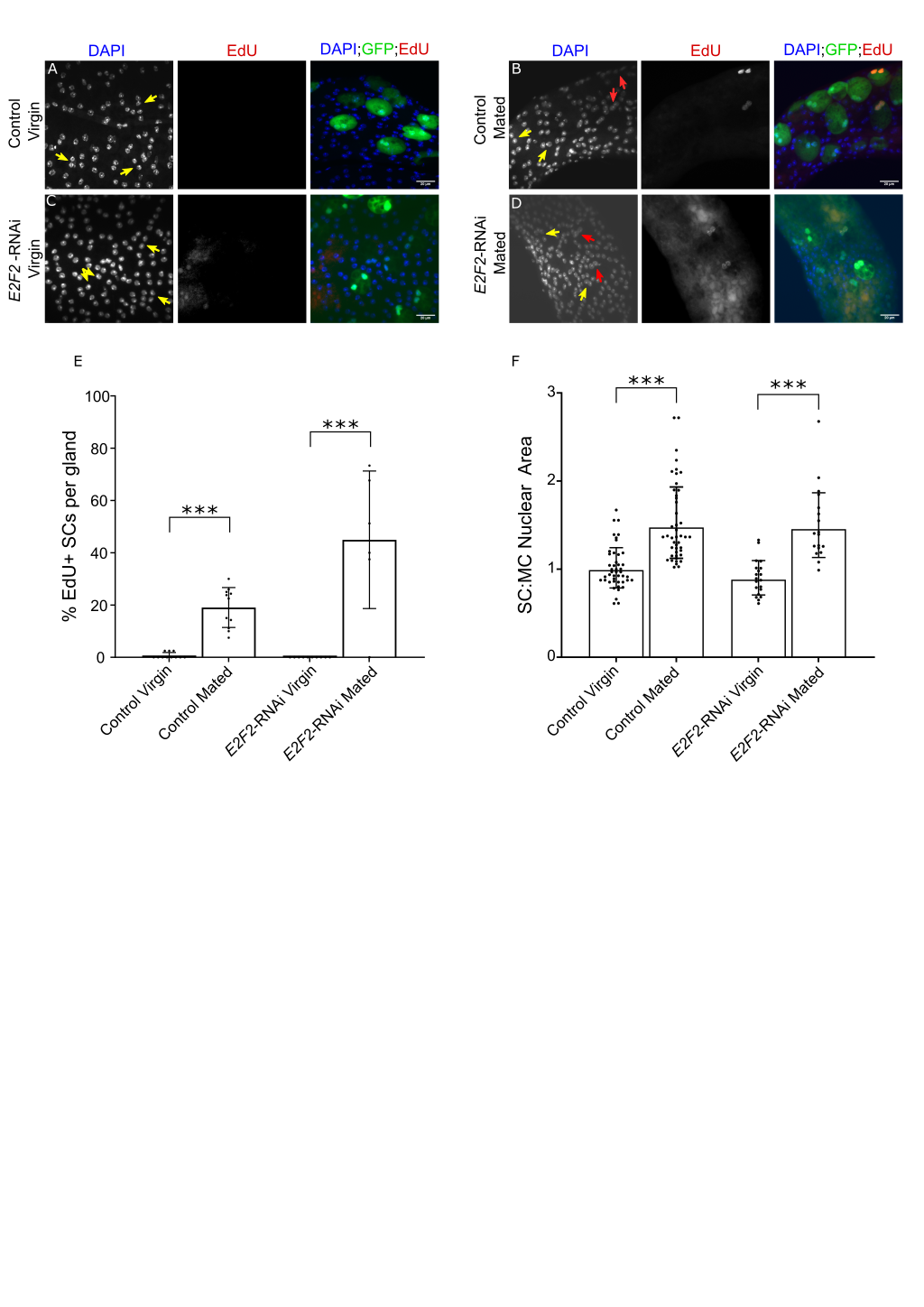
**Figure S4:**

Supplement: S4 Fig — (A-D) Images show distal tip of AGs from 6-day old adult virgin (A, C) or multiply mated (B, D) males expressing nuclear GFP alone (control; this also stains the cytosol; A, B) or in combination with E2F2-RNAi (C, D) in SCs under the control of the esgtsF/O driver and stained for EdU incorporation in SCs. Nuclei are stained with DAPI (blue). Red arrows point to EdU+ pairs of SC nuclei and yellow arrows point to EdU- pairs of SC nuclei for each transgene. (E, F) Bar charts depicting the geometric mean ratio of the size of SC nuclei relative to neighbouring MC nuclei (E) and mean % of EdU+ SCs per gland (F) in virgin and mated flies expressing no other transgene or E2F2-RNAi in SCs. Knocking down E2F2 does not significantly affect SC growth or endoreplication in either virgin or mated males. One-way ANOVA on log-transformed data; Tukey’s HSD post-hoc test (E). Kruskal-Wallis test; Dunn’s post-hoc test (F). n≥18 (E); n≥6 (F). 0.0001<***p≤0.001. (DOCX) [file pgen.1010815.s004.docx]
